# A Major Gene for Bovine Ovulation Rate

**DOI:** 10.1371/journal.pone.0129025

**Published:** 2015-06-05

**Authors:** Brian W. Kirkpatrick, Chris A. Morris

**Affiliations:** 1 Department of Animal Sciences, University of Wisconsin-Madison, Madison, WI, United States of America; 2 AgResearch, Ruakura Research Centre, PB 3123, Hamilton, New Zealand; INRA, FRANCE

## Abstract

Half-sib daughters sired by a bull believed to be a carrier of a major gene for high ovulation rate were evaluated for ovulation rate and genotyped in an effort to both test the hypothesis of segregation of a major gene and to map the gene’s location. A total of 131 daughters were produced over four consecutive years at a University of Wisconsin-Madison research farm. All were evaluated for ovulation rate over an average of four estrous cycles using transrectal ultrasonography. The sire and all daughters were genotyped using a 3K SNP chip and the genotype and phenotype data were used in a linkage analysis. Subsequently, daughters recombinant within the QTL region and the sire were genotyped successively with 50K and 777K SNP chips to refine the location of the causative polymorphism. Positional candidate genes within the fine-mapped region were examined for polymorphism by Sanger sequencing of PCR amplicons encompassing coding and 5’ and 3’ flanking regions of the genes. Sire DNA was used as template in the PCR reactions. Strong evidence of a major gene for ovulation rate was observed (p<1x10^-28^) with the gene localized to bovine chromosome 10. Fine-mapping subsequently reduced the location to a 1.2 Mb region between 13.6 and 14.8 Mb on chromosome 10. The location identified does not correspond to that for any previously identified major gene for ovulation rate. This region contains three candidate genes, *SMAD3*, *SMAD6* and *IQCH*. While candidate gene screening failed to identify the causative polymorphism, three polymorphisms were identified that can be used as a haplotype to track inheritance of the high ovulation rate allele in descendants of the carrier sire.

## Introduction

Examples of major genes for ovulation rate and litter size in sheep are well known. Currently six genes have been either mapped or identified in sheep that cause profound differences in phenotype including *BMPR1B* (Booroola) [1], *BMP15* (Inverdale) [2], *GDF9* [3], *B4GALNT2* [4], Fec^X2W^ [5] and Fec^D^ [6]. Characterization of the underlying genes and polymorphisms for several of these [3, 7–11] has led to the determination of the basis for high prolificacy phenotypes in some sheep breeds and has created a new set of candidate genes and gene networks for consideration in examination of prolificacy and ovarian pathology phenotypes in both livestock and humans. Regarding the use of these loci as candidate genes for variation in prolificacy, no less than 29 highly prolific breeds of sheep (in addition to those in which the major genes were originally discovered) and one goat breed have been examined for evidence of polymorphism at these loci [12–22]. In at least thirteen cases, polymorphisms contributing to the high prolificacy phenotype have been identified. Application of these candidate genes to analysis of prolificacy in humans has been more limited [23–25], but in two cases evidence was discovered for association of *GDF9* mutations with familial dizygotic twinning. A more common focus in humans has been the consideration of these loci as potential candidate genes for ovarian pathologies [26–38]. In livestock, understanding the genetic basis for high prolificacy is valuable for animal genetic improvement and management programs and provides basic knowledge that may facilitate modification of fertility and prolificacy in the future.

The current study examines evidence for segregation of a major gene for ovulation rate in a previously described family of cattle whose matriarch had an exceptional record of prolificacy, having produced three sets of triplets [39]. We hypothesize that this exceptional phenotype is due to the effects of a single locus affecting ovulation rate. Gene mapping studies are reported herein that both support this hypothesis and provide localization of the gene to a genomic region of 1.2 Mb on bovine chromosome 10 (BTA10).

## Materials and Methods

The University of Wisconsin-Madison College of Agricultural and Life Sciences Animal Care and Use Committee approved this research.

### Animal Resources

A son (Trio) of a highly prolific cow (Treble), who had produced three sets of triplets during her lifetime [39], was used by artificial insemination to produce 131 daughters from 2008–2011 (33, 55, 28 and 15 in years 2008–2011, respectively) at a University of Wisconsin-Madison research farm. Trio was born in 1996 as part of Treble's second set of triplets (triplet set consisting of Trio and two sisters); Trio's specific breed composition is unknown though his ancestry likely includes Hereford, Holstein, Angus and Jersey breeds. Trio had himself sired daughters that produced twin and triplet births, providing strong prior evidence of transmission of a genetic factor or factors contributing to high prolificacy. Dams were of mixed breed composition (primarily Angus, Hereford and Holstein-Friesian) with some dams being daughters or granddaughters of sires from the USDA Meat Animal Research Center (USMARC) twinning selection study. Twinning rate among dams at the outset of the study was 5%, so USMARC twinner genetics made only a modest contribution to increased ovulation rate in the Trio daughters.

Trio daughters were evaluated for ovulation rate over an average of four estrous cycles from 12–15 months of age by trans-rectal ultrasonography. Estrus was synchronized at initiation of the ovulation rate evaluation by use of a progesterone pessary (eazi-breed CIDR, Zoetis Inc., Florham Park, NJ) for seven days with administration of prostaglandin F2α at the time of CIDR removal. Animals were ultrasonographically scanned at 10–11 day intervals using either an Easi-Scan ultrasound machine with 7.5 MHz probe or an Ibex-ProLite with 6.5 MHz probe. Ovulation rate was determined by counting corpora lutea (CL) during mid-luteal phase of the estrous cycle.

### Genotyping for Genome Scan and Fine-Mapping

Trio and all daughters evaluated for ovulation rate were genotyped with the Illumina (San Diego, CA) Bovine 3K SNP chip to generate genotypes for a within half-sib family linkage analysis. For DNA, a skin biopsy (ear punch) was obtained at birth or blood was drawn from the coccygeal vein from older animals, without anesthesia. DNA was extracted using a standard proteolytic digestion, organic extraction procedure. Subsequently, Trio and three daughters recombinant within the quantitative trait locus (QTL) peak region, along with two daughters each inheriting alternative sire haplotypes across the region, were genotyped with the Illumina Bovine SNP50 (50K) SNP chip to identify marker brackets containing recombination breakpoints and to identify which animals had recombination breakpoints most narrowly bounding a positional candidate gene region. Subsequently, Trio, the two daughters whose recombination breakpoints most narrowly bounded the QTL region, their dams and two Trio daughters each non-recombinant for alternative Trio haplotypes were genotyped with the Illumina BovineHD SNP (777K) chip to further narrow the recombination breakpoints. Samples and SNPs with call rates below 95% were excluded from analyses.

### Statistical Analyses

Paternally inherited haplotypes across autosomal chromosomes were deduced using a Fortran program as described previously [40]. Linkage analysis was conducted by testing association of paternal haplotype for consecutive marker brackets with mean ovulation rate (animal means over 3–5 estrous cycles) using a linear model analysis implemented in R (R project). The model included effects of year of ovulation rate evaluation as a categorical variable and paternally inherited haplotype as a covariate. Results were plotted as minus log_10_ of the p-value versus marker bracket location. Haplotypes for the 50K and 777K data within the QTL peak region were deduced by manual inspection of the genotype data.

### Positional Candidate Gene Analysis

Three positional candidate genes (*SMAD3*, *SMAD6* and *IQCH*) were screened for polymorphisms within their coding regions and 5' and 3' flanking regions (~1 kb) by generation of overlapping PCR amplicons and subsequent Sanger sequencing. Trio DNA was used as template for generation of the PCR amplicons. Sequence traces were visually inspected for putative polymorphisms using Codon Code Aligner software (CodonCode Corporation, Centerville, MA). Putative polymorphisms were validated and linkage phase with the high ovulation rate QTL allele determined by Sanger sequencing of PCR amplicons derived using pooled template DNA from Trio daughters (n = 10) inheriting either high or low ovulation rate Trio haplotypes in the QTL region. Validated polymorphisms were evaluated initially for allele frequency in the Holstein and Angus breeds and also evaluated for functional relevance such as a non-synonymous change in a coding region, alteration of a consensus splice site sequence or alteration of a known regulatory element. Breed allele frequency estimation in the preliminary evaluation was based on Sanger sequencing of PCR amplicons derived from pooled template DNA (n = 20 animals per breed). Relative peak heights from the sequence traces for the alternative alleles were used to estimate allele frequency [41, 42]. Given the typical infrequency of twin and particularly triplet birth within common cattle breeds, only polymorphisms for which the minor allele was in association with the high ovulation QTL allele and had a preliminary estimate of allele frequency <0.20 were considered as potentially useful markers for tracking inheritance of the high ovulation QTL allele. Polymorphisms passing these criteria were more precisely evaluated for allele frequency using individuals from the Angus (n = 40), Hereford (n = 23), Holstein (n = 53), Jersey (n = 48) and Simmental (n = 24) breeds by genotyping with PCR-RFLP (Table 1). All PCR reactions were performed with a touchdown protocol that had an initial annealing temperature of 63°C which was reduced by 0.5°C per cycle until an annealing temperature of 58°C was obtained. Go*Taq* DNA polymerase (Promega Inc., Madison, WI) was used in all reactions per the manufacturer's recommendations. Primer design and expected restriction fragment sizes are indicated in Table 1. Fragment size separation was accomplished by electrophoresis using 4% agarose (1% regular and 3% sieving agarose) gels. Haplotypes were predicted from genotype data using fastPHASE [43] and linkage disequilibrium (LD) was calculated using Haploview [44]. Allele and haplotype frequencies were estimated by counting.

**Table 1:** PCR-RFLP assays.

BTA10 Location (bp)^1^, polymorphism ID	Forward Primer^2^	Reverse Primer	Amplicon Length (bp)	Restriction Enzyme	Fragment size (bp), allele 1	Fragment size (bp), allele 2
**13,663,940 ss1714766385**	tttgCTCAGTAGTTGCGCAGTAcG	AAATTGAGTTGGGGGCTTCC	141 or 145	*Hpy*CH4IV	141, G	122 and 23, GTATG
**14,263,362 ss1714766398**	GGGTCGTGTATCGCACTTTGTT	TTGTCCCTCTTCCCACAGGTAA	307	*Hpy*CH4III	307, A	188 and 119, C
**14,270,483 ss1714766400**	gTTTGCTCCCAAGAAAGACAAGAAcAT	ATGGAGCTTGGCACACAACC	128	*Nla*III	128, A	102 and 26, G

^1^ Bovine genome assembly UMD3.1

^2^ Lower case letters denote non-complementary bases

## Results and Discussion

**Fig 1:**
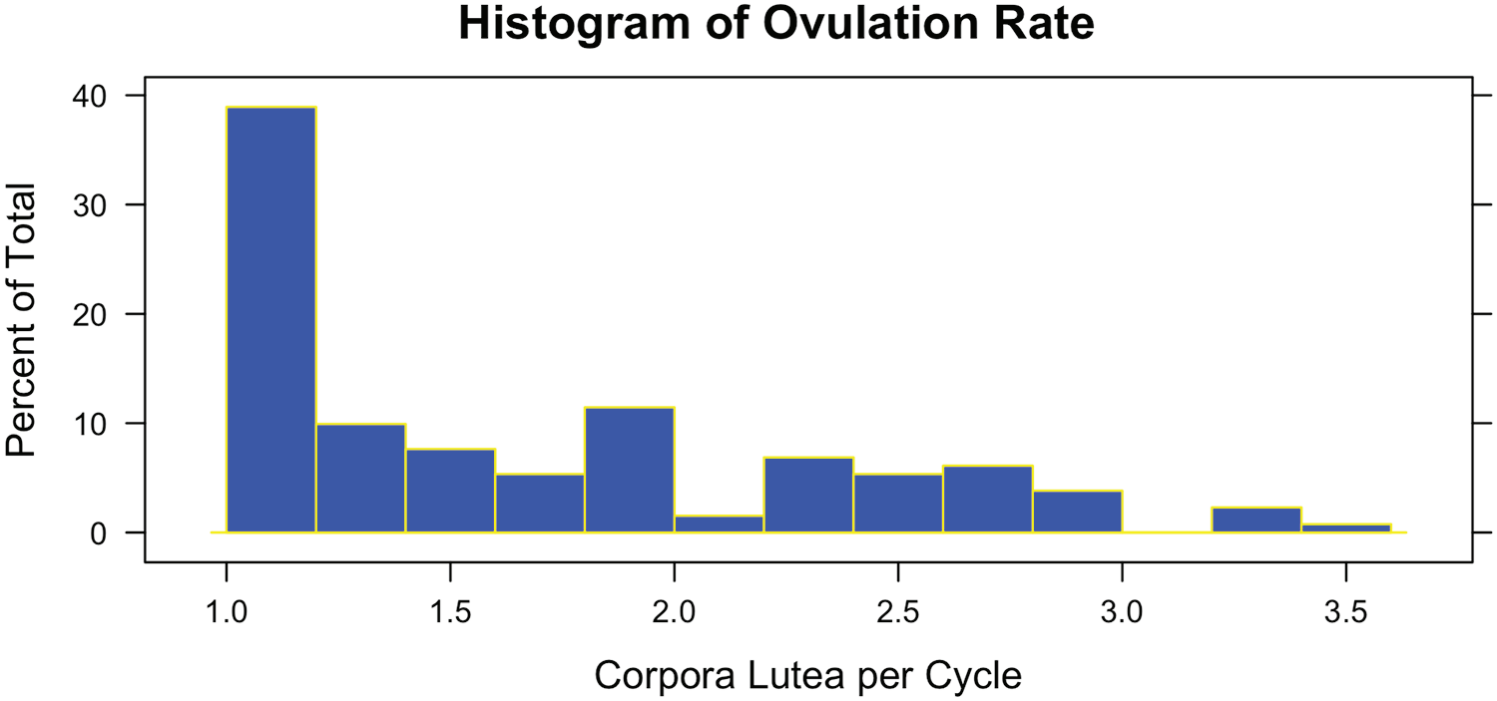
Distribution of animal ovulation rate means. Ovulation rate was determined by count of corpora lutea over four estrous cycles on average.

Average ovulation rate across all heifers was 1.67 CL per cycle with a range of one to 3.5 CL per cycle for animal means. Considering individual estrous cycles, the range of observations was from one to five CL per cycle. The distribution of ovulation rate means was not unambiguously bi-modal (Fig 1).

**Fig 2:**
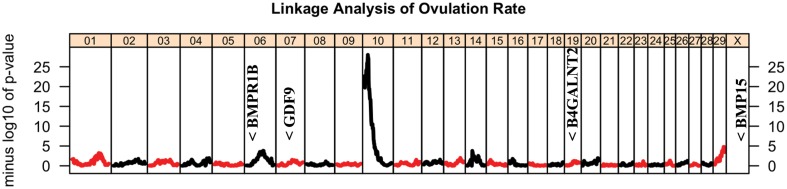
Within half-sib family linkage analysis for ovulation rate. Minus log_10_ of the p-value from the test of association of paternally inherited haplotype with ovulation rate is shown on the y-axis and the x-axis corresponds to marker location. Each vertical panel corresponds to one chromosome. Approximate locations of known major genes for ovulation rate in sheep are indicated.

**Fig 3:**
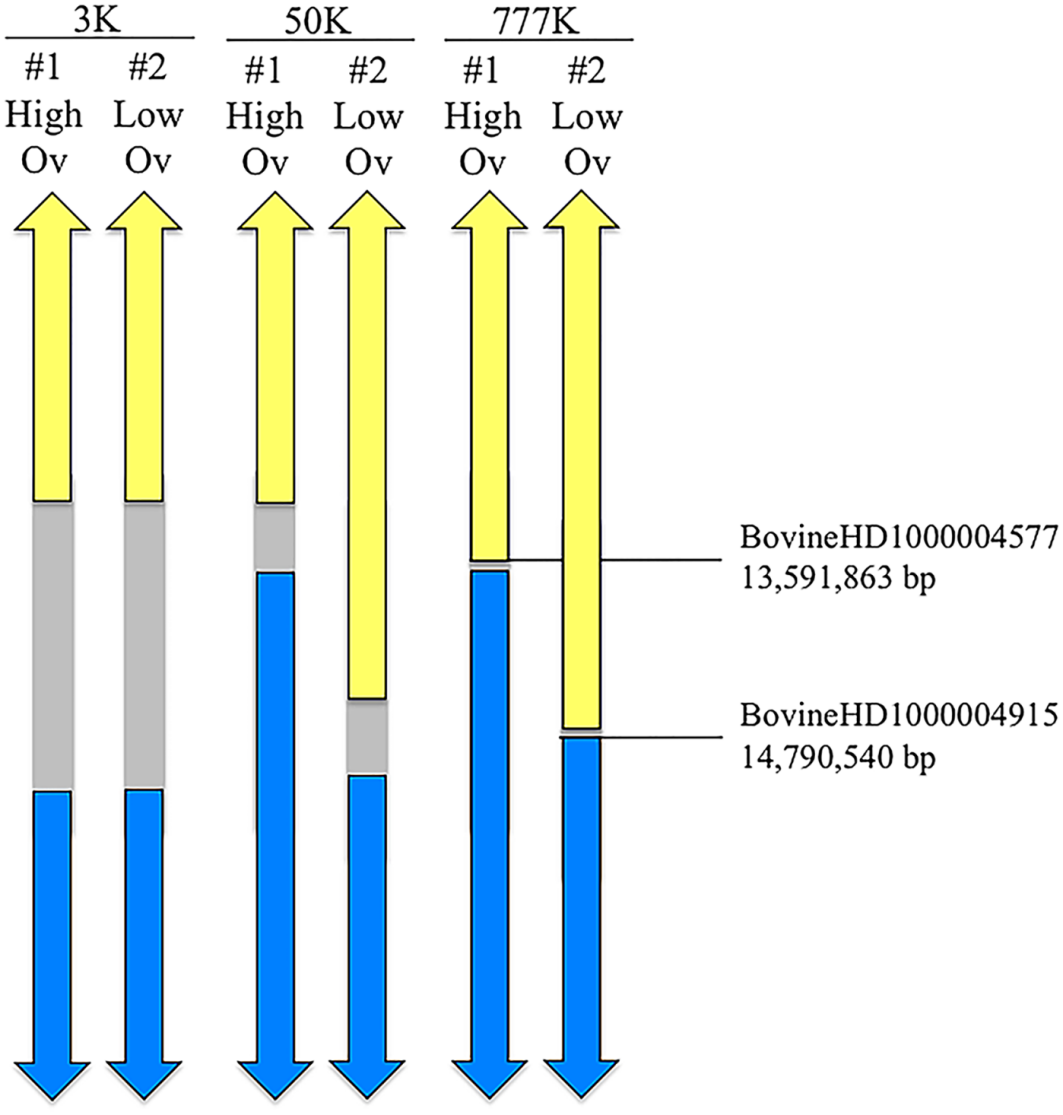
Results for fine-mapping based on the two Trio offspring (denoted #1 and #2) whose recombination breakpoints most narrowly bound the candidate gene region. Upward and downward pointing arrows denote extension of the indicated haplotype to the centromeric and telomeric ends of the chromosome, respectively. Areas in grey denote regions of unknown haplotype. High density genotyping resulted in a narrowing of the candidate gene region to a length of 1.2 Mb bounded by markers BovineHD1000004577 and BovineHD1000004915, as indicated.

Linkage analysis provided strong evidence (p<1x10^-28^) for segregation of a major gene for ovulation rate (Fig 2). Subsequent analysis of 50K SNP data for recombinant Trio daughters narrowed the region to between 13.2 and 15.1 Mb on BTA10 and identified the two daughters whose recombination breakpoints most narrowly bounded the candidate gene region. Analysis of HD genotype data for these individuals further narrowed the location of the causative polymorphism to a region between 13.6 and 14.8 Mb on BTA10 (Fig 3) which contains eight characterized genes and six uncharacterized loci. The most likely candidate genes within this region are SMAD family member 3 (*SMAD3)*, SMAD family member 6 (*SMAD6)* and IQ motif containing H (*IQCH)*, at approximately 13.7 Mb, 13.9 Mb and 14.2 Mb, respectively.

Analysis of coding and 5’ and 3’ flanking regions of candidate genes within this region revealed a total of 30 polymorphisms (Table 2), but none were rare and additionally none were alterations of coding or regulatory sequences. However, three polymorphisms with minor alleles in association with the high ovulation rate allele (Table 3) comprised a haplotyping system with a relatively rare haplotype in association with the high ovulation rate allele (Table 4). The haplotype associated with increase ovulation rate has the insertion allele at locus 13663941, the A allele at locus 14263362 and the A allele at locus 14270483. Linkage disequilibrium between these three loci was variable across breed (Table 5) with a tendency, particularly in the beef breeds, for lower LD between the first and second and first and third loci, and higher LD between the second and third loci, in keeping with the close proximity of the latter two. This relatively infrequent haplotype has utility for tracking inheritance of the high ovulation rate allele in Trio descendants. Frequency of this haplotype ranged from zero to 0.064 within breed (Table 4).

**Table 2:** Validated polymorphisms within positional candidate genes.

				Allelic association with paternal haplotypes		Frequency of allele associated with high ovulation paternal haplotype^2^	
Candidate Gene	dbSNP ss#	Type	Location^1^ (UMD3 BTA10)	High ovulation haplotype	Normal ovulation haplotype	Holstein	Angus
***SMAD6***	ss1714766371	SNP	13504421	T	C	1.000	1.000
***SMAD6***	ss1714766372	SNP	13504699	A	T	0.959	1.000
***SMAD6***	ss1714766373	SNP	13504752	C	T	0.949	0.926
***SMAD6***	ss1714766374	SNP	13506509	T	G	1.000	0.951
***SMAD6***	ss1714766375	SNP	13514898	C	T	1.000	0.764
***SMAD6***	ss1714766376	9 bp indel	13516530	AAGAGCCCGA	A	0.436	0.309
***SMAD6***	ss1714766377	Indel	13516622	A	AC	1.000	0.942
***SMAD6***	ss1714766378	SNP	13518467	C	T	0.798	0.899
***SMAD6***	ss1714766379	SNP	13518480	A	G	1.000	0.907
***SMAD6***	ss1714766380	SNP	13518558	C	A	0.822	0.797
***SMAD6***	ss1714766381	1 bp indel	13518552	A	AG	1.000	0.975
***SMAD6***	ss1714766382	1 bp indel	13519035	A	GA	0.989	0.914
***SMAD6***	ss1714766383	SNP	13519130	C	T	0.982	0.939
***SMAD6***	ss1714766384	SNP	13583995	G	A	0.684	0.858
***SMAD6***	ss1714766385	4 bp indel	13663940	GTATG	G	0.116	0.043
***SMAD3***	ss1714766386	SNP	13858002	C	T	1.000	0.986
***SMAD3***	ss1714766387	SNP	13959936	C	T	0.484	ND
***SMAD3***	ss1714766388	SNP	13960164	C	T	0.542	0.068
***SMAD3***	ss1714766389	SNP	13960227	T	C	0.605	0.091
***SMAD3***	ss1714766390	SNP	13980613	C	G	0.407	0.088
***IQCH***	ss1714766391	SNP	14075120	T	C	0.305	0.117
***IQCH***	ss1714766392	SNP	14097896	T	C	0.979	ND
***IQCH***	ss1714766393	SNP	14184550	G	A	0.951	0.916
***IQCH***	ss1714766394	SNP	14204481	A	G	1.000	0.966
***IQCH***	ss1714766395	SNP	14240496	A	T	1.000	0.971
***IQCH***	ss1714766396	SNP	14263275	C	T	0.307	0.113
***IQCH***	ss1714766397	SNP	14263281	A	T	0.472	0.211
***IQCH***	ss1714766398	SNP	14263362	A	C	0.000	0.000
***IQCH***	ss1714766399	SNP	14263404	C	G	0.328	0.123
***IQCH***	ss1714766400	SNP	14270483	A	G	0.099	0.000

Location in basepairs on BTA10 from bovine genome assembly UMD 3.1. Preliminary estimate of allele frequency based on electropherogram peak heights of alternative alleles using pooled DNA samples. ND = not determined.

**Table 3:** Frequency of allele associated with high ovulation rate by breed for three polymorphisms.^1^

	Number of animals	ss1714766385 (+)^2^	ss1714766398 (A)	ss1714766400 (A)
**Angus**	40	0.175 ± 0.060	0.077 ± 0.042	0.113 ± 0.050
**Hereford**	23	0.196 ± 0.083	0.109 ± 0.065	0.087 ± 0.059
**Holstein**	53	0.030 ± 0.024	0.048 ± 0.030	0.057 ± 0.033
**Jersey**	48	0.022 ± 0.022	0.021 ± 0.021	0.042 ± 0.029
**Simmental**	24	0.136 ± 0.073	0.130 ± 0.072	0.083 ± 0.059

^1^ Allele frequency estimated by genotyping individual animals and counting alleles.

^2^ "+" denotes the allelic form containing an insertion of TATG, as indicated in Table 2. Loci are designated by their dbSNP submission number, as indicated in Table 2.

**Table 4:** Frequency of haplotypes by breed.^1,2^

Breed	Number of animals	+_A_A	+_C_G	-_A_A	-_A_G	-_C_A	-_C_G
**Angus**	40	0.063 ± 0.038	0.113 ± 0.050	0.025 ± 0.025	—	0.025 ± 0.025	0.775 ± 0.066
**Hereford**	23	—	0.136 ± 0.072	0.045 ± 0.043	0.045 ± 0.043	—	0.773 ± 0.087
**Holstein**	53	0.019 ± 0.019	—	0.019 ± 0.019	—	0.019 ± 0.019	0.943 ± 0.033
**Jersey**	48	0.020 ± 0.021	—	—	—	0.020 ± 0.021	0.960 ± 0.029
**Simmental**	24	0.064 ± 0.052	0.085 ± 0.059	—	0.064 ± 0.052	—	0.787 ± 0.087

^1^ Haplotypes denoted as “ss1714766385 allele_ ss1714766398 allele _ ss1714766400 allele”. "+" denotes the allelic form containing an insertion of TATG, as indicated in Table 2.

^2^ The "+_A_A" haplotype is associated with increased ovulation rate within the Trio family.

**Table 5:** Linkage disequilibrium (r^2^) between markers.^1^

Breed	Number of animals	ss1714766385 and ss1714766398	ss1714766385 and ss1714766400	ss1714766398 and ss1714766400
**Angus**	40	0.13	0.10	0.72
**Hereford**	23	0.03	0.18	0.39
**Holstein**	53	0.58	0.19	0.50
**Jersey**	48	1.00	1.00	0.48
**Simmental**	24	0.03	0.16	0.46

^1^ Markers designated by their location in basepairs.

Ovulation rate for daughters inheriting alternative haplotypes from Trio were 2.19 ± 0.57 ova for carriers (n = 68) of the high allele versus 1.11 ± 0.22 ova for non-carriers (n = 63). Classification was based on BTA10 haplotypes for non-recombinants within the QTL peak region and the above-described three polymorphism haplotyping system for daughters recombinant within the QTL peak region.

Strong evidence is presented for segregation of a single gene for high ovulation rate within the Treble/Trio extended family. However, the causative polymorphism and gene remain uncertain. Three positional candidate genes residing within the positional candidate gene region were examined for polymorphisms within the coding regions and proximal 5’ and 3’ flanking regions, but no likely causative polymorphism was identified. This suggests that the causative polymorphism may be within a regulatory sequence, and more distant from the gene than the 1 kb flanking regions considered in the candidate gene screening. Alternatively, the causative polymorphism may be associated with a gene other than the three candidates considered. Initial efforts (unpublished) at a more comprehensive screening of the positional candidate gene region were dissatisfying owing both to the inability to capture and sequence the entire positional candidate gene region and to the presence of duplications within the positional candidate gene region which complicate the identification of true polymorphisms. To overcome these issues individuals homozygous for the high ovulation haplotype are being created through carrier x carrier matings to serve as a source of DNA for a *de novo* sequence assembly which is expected to provide a comprehensive screening of the candidate region.

The genomic region harboring the high ovulation rate polymorphism does not correspond to the location that should be the homologous location of any major genes for ovulation rate or litter size previously identified in sheep (Fig 2). Likewise, while QTL for twinning rate [45] or ovulation rate [46] in cattle have been mapped to the same chromosome, and litter size QTL in mice have been mapped to a chromosome with partial correspondence to the same gene region [47], in neither case was the gene location included within the QTL confidence interval; further, for the mouse the QTL region corresponded primarily to a different bovine chromosome. The closest correspondence in location is for an association with litter size in pigs (number born, number born alive) in which the QTL location at approximately 173.6 Mb on swine chromosome 1 corresponds to the location of *FEM1B*, mentioned above [48]. However, the failure to also identify an ovulation rate QTL at the corresponding genomic location within the same swine population suggests the litter size QTL may be attributable to something other than an ovulation rate mechanism [49] while the gene mapped here clearly affects ovulation rate.

The fact that the gene mapped here does not correspond to any previously identified major gene for ovulation rate does not necessarily imply that the gene is unrelated to a previously identified major gene. Specifically, two of the positional candidate genes considered, *SMAD3* and *SMAD6*, are part of the TGFß signaling pathway which is relevant to one of the known major genes, *GDF9*. Given the intermediary role of the Smad-3 protein in the high ovulation rate effects of *GDF9* variants in sheep, *SMAD3* is a strong positional candidate gene. The *SMAD6* gene product, Smad-6, is an inhibitor in this signaling pathway that works in an opposing manner to Smad-2 and Smad-3 and is therefore also a strong candidate gene.

Two other loci which are unrelated to the TGFß system are either within the candidate gene region or in very close proximity and merit consideration. *IQCH*, located roughly in the middle of the positional candidate region, has been linked with age at menarche in human females in a whole genome association study meta-analysis [50] and was considered here in screening for polymorphisms. A second locus, *FEM1B*, has been associated with polycystic ovary syndrome in humans [51, 52]. Its location just distal (300 kb) of the positional candidate gene region excluded it from the polymorphism screening effort; however, given the close proximity of *FEM1B* to the region, it is possible that a regulatory mutation within the candidate gene region could impact its expression. In this regard, reference to a positional candidate gene region is probably a misnomer, and reference should instead be to a positional candidate polymorphism region.

In subsequent work, the ovulation rate associated with the high allele has been higher than reported here with an average of four ova for carriers versus one for non-carriers (unpublished). Multiple causes may contribute to the lesser difference between carrier and non-carriers reported here and include possible increase in ovulation rate with age, as has been observed previously for polygenic ovulation rate [53], and possible inaccuracy in ultrasonic evaluation (BWK) leading to understatement of true ovulation rate for carrier females. Corpora lutea diameter in carrier females is significantly smaller than in non-carriers which may have led to some errors in corpora lutea count in the initial group of females (average count rose from 1.51± 0.38 to 1.88 ± 0.81 CL from the first to third years of evaluation). One might expect that the presence of MARC twinner genetics within the herd would contribute to an overstatement of ovulation rate, however that contribution would be equivalent for both carrier and non-carrier females, apart from interaction between loci, and MARC twinner genetics comprised a small part of the genetic make-up of the herd as evidenced by the modest twinning rate within the herd in the year preceding the onset of this study (~5%). Anecdotal evidence of the understatement of ovulation rate comes also from an abortion rate among carrier females in the UW herd in excess of 50% with the observable late term abortions typically being triplet and quadruplet pregnancies. For practical application of this allele it will be necessary to modulate ovulation rate. Nutritional, hormonal, or physical approaches may be ways to achieve this. The increase of ovulation rate in response to increased energy and/or protein in the diet of swine and sheep is well known [54] and the opposite treatment might reduce ovulation rate for this allele. Regarding hormonal treatment, research with ewes possessing the *BMPR1B* allele has demonstrated the ability to reduce ovulation rate through administration of estradiol [55], presumably due to negative feedback effects on follicle stimulating hormone release from the pituitary. Finally, selective ablation of follicles by ultrasound-guided follicular aspiration would be a means of specifically reducing ovulation rate to two ova, preferentially bi-lateral ovulation, maximizing chances for twin pregnancy [56].

Many unanswered questions, besides what are the polymorphism and gene responsible, are raised by this work including what phenotype will be associated with a homozygous genotype, what are the effects of this mutation on male fertility, are there effects of the mutation on reproductive longevity, what is the physiological mechanism for this mutation’s effect on ovulation rate, and are there effects of this mutation on non-reproductive characteristics? Some of the major genes for ovulation rate in sheep (*BMP15*, *GDF9*) are known to cause infertility in the homozygous genotype [3, 7], for some alleles, while others (*BMPR1B*) lead to even higher ovulation rate in the homozygote versus the carrier [57]. Regarding male fertility and reproductive longevity, there are no reports from previous work with major genes for ovulation rate in sheep that suggest effects on these characteristics. Finally, global gene expression analysis within relevant tissue or cell types, such as granulosa cells, should provide both a test of hypotheses concerning differences in expression of positional candidate genes as well as a picture of downstream effects of the mutation on expression of other genes, helping determine how this mutation ultimately causes alteration of ovulation rate.

Regardless of whether this polymorphism can be practically applied or not, identification of the gene and causative polymorphism will advance understanding of the genetic basis for variation in ovulation rate. This information will both deepen understanding of the mechanism underlying variation in ovulation rate and provide a new candidate gene to potentially explain genetic variants for ovulation rate, multiple births and ovarian pathologies.

## Supporting Information

S1 File Dataset S1. Phenotype data used for linkage analysis. File includes animal ID, average ovulation rate and birth year.
(TXT)

S2 File Dataset S2. Genotype data used for linkage analysis. File includes animal ID, SNP ID and location, and numerically coded SNP alleles.
(ZIP)
